# Regime shifts and climate responses of alpine grasslands in Gannan Prefecture, China

**DOI:** 10.3389/fpls.2026.1851128

**Published:** 2026-06-26

**Authors:** Qingqing Hou, Yanrong Tan, Quanlin Ma, Li Zhu, Wenye Chen, Danhui Bing, Binjie Wang, Guohong Wu

**Affiliations:** 1 Gansu Academy of Forestry, Lanzhou, China; 2 Key Laboratory of State Forestry and Grassland Administration on “Mountain-River-Forest-Farmland-Lake-Grassland-Desert” System Governance, Lanzhou, China; 3 Gansu Gahai Wetland Ecosystem Positioning Observation and Research Station, Luqu, China

**Keywords:** alpine grassland, climate change, ecological resilience, regime shift, spatiotemporal

## Abstract

Ecological regime shifts and resilience are critical for maintaining the stability and sustainability of grassland ecosystems under climate change. However, existing studies on alpine grasslands primarily rely on single indicators and rarely integrate ecosystem structure and function to comprehensively characterize regime dynamics. Taking the alpine grasslands of Gannan Prefecture as a case study, this study quantitatively analyzed long-term grassland dynamics from three dimensions: grassland area, vegetation structure (fractional vegetation cover, FVC), and ecosystem function (net primary productivity, NPP). The Sequential t-test Analysis of Regime Shifts (STARS) method was employed to identify regime shifts, while the “ball-and-cup” model was used to quantify ecological resilience, and the responses of regime dynamics to climate factors were further explored. The results showed that: (1) grassland area decreased by approximately 0.5% during 1995–2024, primarily converting to forest, mainly distributed in Zhuoni, Diebu, and Zhouqu counties, with a total conversion area of approximately 88,600 hm^2^; (2) Analyses based on FVC and NPP identified two major regime shifts in the alpine grassland ecosystem during 2001–2023, and the detected transition periods showed certain similarities. The strongest FVC-based regime shift occurred in 2018, whereas the most pronounced NPP-based shift occurred around 2006; (3) Ecological resilience exhibited a decreasing trend during the study period, decreasing by 16.03% based on FVC and by 3.85% based on NPP; (4) Furthermore, regime shifts were closely associated with both precipitation and temperature. FVC-based regime shifts suggested a possible lagged response to precipitation, whereas NPP-based regime shifts exhibited relatively synchronous patterns with temperature variability. By integrating structural and functional indicators, this study provides a more comprehensive framework for understanding alpine grassland regime dynamics and resilience under climate change. The findings provide a theoretical basis and methodological references for grassland conservation, ecological restoration, and sustainable ecosystem management in ecologically fragile alpine regions.

## 1 Introduction

Regime refers to a state in which the structural characteristics and ecological functions of an ecosystem remain relatively stable over specific spatial and temporal scales. When an ecosystem transitions from one stable state to another, it often exhibits a nonlinear abrupt process, commonly referred to as a regime shift (Lu et al., 2025; Vasilakopoulos et al., 2017). External disturbances can trigger sudden changes in ecosystem states, potentially driving ecosystems into undesirable states from a management perspective. Even without direct human intervention, long-term environmental changes (e.g., climate change, habitat fragmentation) may gradually exceed system thresholds, ultimately triggering abrupt regime shifts. The newly established states following such transitions typically generate adverse effects, as the beneficial services provided by the original ecosystem decline significantly (Walker et al., 2002; Holling and Meffe, 1996). For instance, eutrophication in freshwater lakes is frequently associated with biodiversity collapse. Once a regime shift occurs, restoring the ecosystem to its original state is typically challenging, costly, and sometimes irreversible (Peterson et al., 1998; Scheffer et al., 2001). Therefore, understanding ecological regime dynamics and their driving mechanisms is essential for ecosystem restoration, conservation, and sustainable management.

In recent years, increasing attention has been paid to ecological resilience because of its close relationship with regime dynamics. Resilience refers to an ecosystem’s capacity to absorb disturbances while maintaining its structural and functional stability (Walker et al., 2004; Holling, 2001). Ecosystems with high resilience generally exhibit greater resistance to external perturbations, whereas reduced resilience increases ecosystem sensitivity and vulnerability to even minor disturbances (Yang et al., 2021a). For instance, a study combining field investigations and literature analysis of 46 lakes in the Yangtze River Basin explored total phosphorus thresholds for transitions between clear-water and turbid-water states (Wang et al., 2014). Chu et al. (2026) analyzed stoichiometric homeostasis to investigate regime shifts and their driving mechanisms in a representative inland river wetland under grazing pressure and further identified the non-equilibrium thresholds. However, traditional experimental and field observation approaches have certain limitations in long-term and large-scale regime shift research (Zhang et al., 2017).

With the rapid development of remote sensing technology, multi-source long-term datasets now provide effective tools for quantifying ecosystem regime dynamics and resilience variations across large spatial scales (Yang et al., 2021a). Consequently, remote sensing approaches have been increasingly applied in related studies (Gilarranz et al., 2022; Liu et al., 2025a; Yang, 2019). Existing studies have demonstrated the effectiveness of remote sensing in detecting regime shifts in different ecosystems. For example, Gilarranz et al. (2022) used time-series trophic state indices derived from satellite imagery to conduct a global assessment of regime shifts and stability in lake productivity across 1,015 lakes worldwide. Liu et al. (2025a) used connectivity indices and spatial vegetation pattern indices to explore the effectiveness of hydrological connectivity as an early warning indicator for ecosystem degradation and to identify its critical thresholds. Yang (2019) applied a modified vegetation-water index to detect wetland regime shifts in the Beijing-Tianjin-Hebei region by analyzing temporal variations, spatial intensity distributions, and critical transition years, and further explored resilience variation patterns based on these shifts.

Although previous studies have established an important foundation for understanding ecosystem stability and resilience, several limitations still remain. First, current studies mainly concentrate on wetland and marine ecosystems, whereas relatively limited attention has been paid to grassland ecosystems. Second, most existing studies rely on single indicators, such as vegetation cover or productivity, to characterize ecosystem regime dynamics. However, single-indicator approaches may not comprehensively capture the characteristics of regime shift processes. Therefore, integrating multiple ecological indicators to jointly characterize ecosystem regime dynamics and resilience remains an important research challenge.

As an important ecological barrier in the upper reaches of the Yellow River and the Yangtze River, Gannan Prefecture is a nationally ecological functional area and an integral part of the Qinghai-Tibet Plateau (Zhou et al., 2025). This region possesses abundant grassland resources that are highly sensitive to climate change and human activities. Under the combined influence of multiple factors, particularly global climate change, the ecosystem of Gannan Prefecture has experienced a series of ecological problems, including grassland degradation and biodiversity loss (Su et al., 2025). Therefore, exploring the responses of alpine grassland regime shifts and ecological resilience to climate change is essential for understanding grassland succession processes and supporting regional ecological restoration and sustainable management. However, studies on regime shifts and resilience dynamics in the grassland ecosystems of Gannan Prefecture remain limited.

To address these research gaps, this study focuses on the alpine grasslands of Gannan Prefecture and aims to answer the following scientific questions: (1) how have land use patterns changed over the long term, and what are the primary land use types for grassland inflows and outflows? (2) what are the spatiotemporal characteristics of regime shifts and ecological resilience in alpine grasslands? and (3) how do regime dynamics and resilience respond to climate change? To address these questions, this study first quantitatively analyzed long-term land use changes in alpine grasslands. Subsequently, the STARS method was employed to identify regime shifts, and the “ball-and-cup” model was applied to quantify ecological resilience. Finally, the relationships between regime dynamics and climatic factors were explored to elucidate the underlying driving mechanisms.

Unlike previous studies that relied on single indicators, this study integrates structural (FVC) and functional (NPP) indicators to jointly characterize alpine grassland regime dynamics and resilience, providing a more comprehensive framework for understanding ecosystem stability. The combined use of FVC and NPP allows not only the detection of changes in vegetation cover and ecosystem productivity, but also the comparison of their differential responses to climate change and ecosystem stability. This approach helps reveal whether vegetation “greening” necessarily corresponds to enhanced ecosystem resilience, thereby providing a more comprehensive understanding of ecosystem regime dynamics than single-indicator approaches. By linking regime shifts with climatic drivers, this study not only advances the understanding of ecosystem responses to climate change in alpine regions but also offers methodological support for ecosystem monitoring and management in ecologically fragile areas.

## 2 Materials and methods

### 2.1 Study region

**Figure 1:**
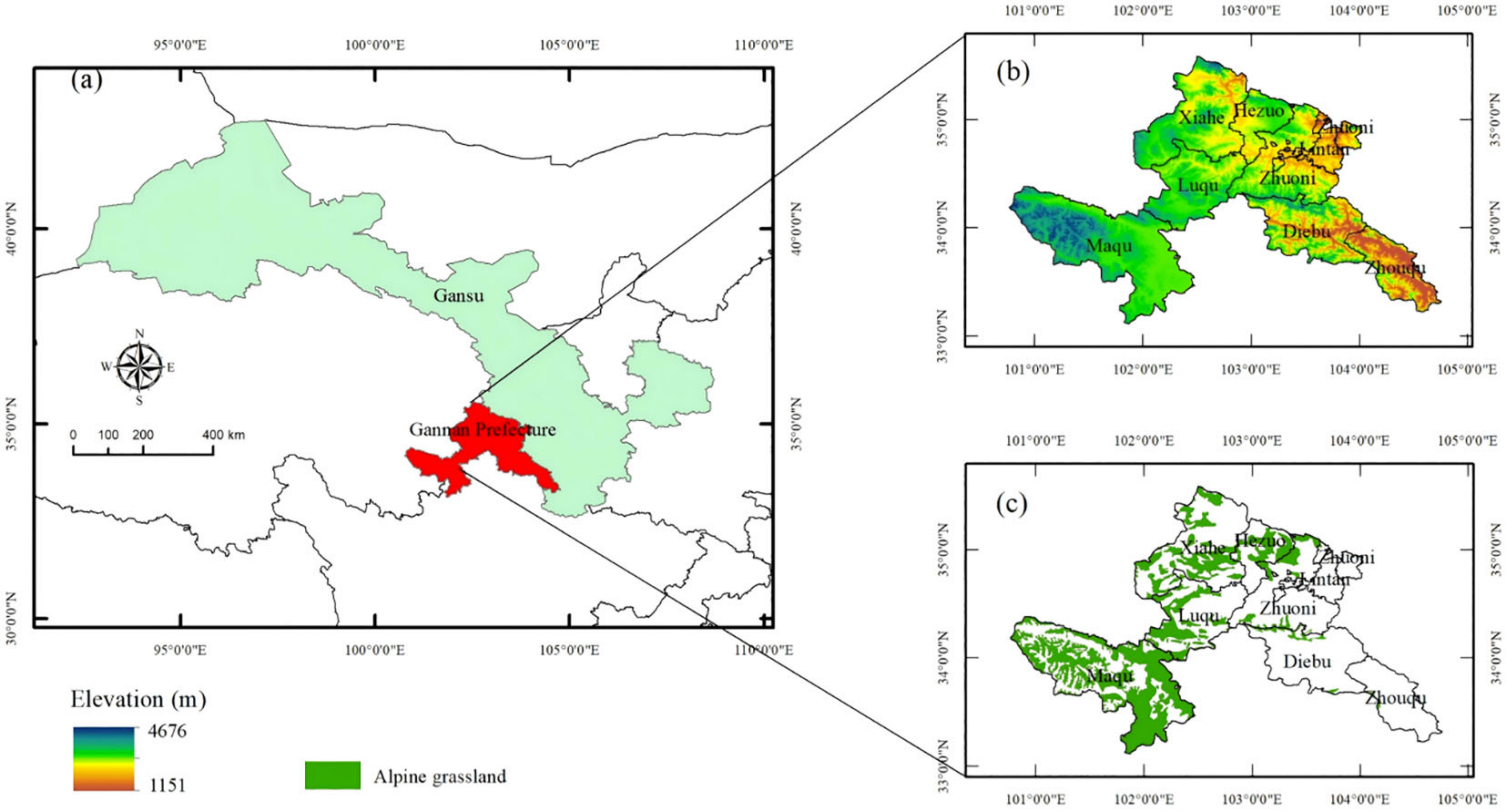
Study area overview map [Geographical location **(a)**, altitude **(b)**, and the spatial distribution of alpine grassland types **(c)**].

Gannan Prefecture, located in southwestern Gansu Province (33°06′N~35°34′N; 100°46′E~104°44′E), is situated in the transitional zone between the Qinghai-Tibet Plateau and the Loess Plateau. It serves as an important water conservation and recharge area for both the Yellow River and Yangtze River systems and functions as a key convergence zone of three major natural regions (Wu et al., 2024; Zhang, 2025a). The prefecture covers approximately 45,000 km^2^ and administers seven counties and one city (**Figure 1**). The region is characterized by complex topography, with generally higher elevations in the northwest and lower elevations in the southeast. Multiple landforms, including plains, hills, mountains, and tablelands, are distributed throughout the region, with an average elevation ranging from 3,000 to 3,500 m. The climate is classified as a plateau continental climate, characterized by an annual average temperature of approximately 1.7 °C and annual mean precipitation ranging from 400 to 800 mm. Variation in topography and climatic zones result in pronounced spatial climatic heterogeneity and significant vertical temperature gradients (Su et al., 2025; Chen et al., 2025). Land cover types in the study area primarily include grassland, forest, shrubland, and wetland. Grassland covers approximately 27,200 km^2^, accounting for 71.08% of the total area, making the region an important ecological security barrier and biodiversity conservation area in northwestern China (Zhang et al., 2025b).

### 2.2 Data sources and preprocessing

Land cover data for Gannan Prefecture in 1995, 2005, 2015, and 2024 were obtained from the China Land Cover Dataset (CLCD) provided by Wuhan University, with a spatial resolution of 30m (Yang and Huang, 2025). Grassland types, fractional vegetation cover (FVC), net primary productivity (NPP), digital elevation model (DEM), and meteorological data were sourced from authoritative institutions. Detailed information on data sources and descriptions is provided in **Table 1**.

**Table 1:** Major data sources.

Dataset	Product/version	Data type	Data time	Spatial resolution	Data source	Access date
China Land Cover Dataset (CLCD)	Version 1.0.4	Raster	1995, 2005, 2015, 2024	30m	Zenodo platform (https://zenodo.org/records/12779975)	Nov, 2025
Digital Elevation Model (DEM)	SRTM DEM V4.1	Raster	2024	90m	Resources and Environmental Science Data Platform (https://www.resdc.cn/)	Jan, 2021
Temperature and precipitation	Temperature and precipitation dataset for China	Raster	2001–2023	1km	National Earth System Science Data Center (https://www.geodata.cn/aboutus.html)	Mar, 2026
Fractional vegetation cover (FVC)	China regional fractional vegetation cover dataset	Raster	2001–2023	250m	National Tibetan Plateau Data Center (https://data.tpdc.ac.cn/)	Nov, 2025
Net primary productivity (NPP)	MOD17A3HGF.061	Raster	2001–2023	1km	Resources and Environmental Science Data Platform (https://www.resdc.cn/)	Nov, 2025
Vegetation type map	1:1 million Vegetation Map of China	Raster	–	–	National Tibetan Plateau Data Center (https://data.tpdc.ac.cn/)	Sep, 2025

To ensure spatial consistency among datasets with different original spatial resolutions, all raster datasets were resampled and spatially aligned to a unified 30 m grid using the “Resample” tool in ArcGIS 10.8. To characterize long-term land use change (LUCC), four periods of land use data (1995, 2005, 2015, and 2024) were used to capture decadal-scale changes in grassland area and land conversion patterns. In contrast, regime shift and ecological resilience analyses were conducted using continuous annual FVC and NPP datasets from 2001 to 2023 derived from MODIS products. To ensure consistency in ecosystem analysis, regime shift detection and ecological resilience calculations were performed only within the alpine grassland distribution area defined by the 1:1 million Vegetation Map of China.

### 2.3 Methods

#### 2.3.1 Land use transition matrix

To characterize the transition relationships among land use types across different periods, this study employs the land use transition matrix to quantify the inflow and outflow of various land use types between two time points, thereby revealing the quantitative characteristics and evolutionary trends of land use changes in the study area (Liu and Song, 2025b). The mathematical model is expressed as follows:


$$L_{ij}=\left[\begin{array}{cccc} L_{11} & L_{12} & \ldots & L_{1n}\\ L_{21} & L_{22} & \ldots & L_{2n}\\ \ldots & \ldots & \ldots & \ldots \\ L_{n1} & L_{n2} & \ldots & L_{mn} \end{array}\right]$$

In the formula, n denotes the number of land use types, *i* and *j* represents the land use type at the initial and final stage, respectively. *L_ij_* denotes the area converted from land use type *i* to type *j*.

#### 2.3.2 Regime shift detection

In the long-term evolution of ecosystems, the combined effects of multiple factors, such as human activities and climate change, often lead to ecosystem changes (Yang et al., 2021a; Li, 2017). Regime shifts from 2001 to 2023 were analyzed to identify regime shift years and spatial distribution patterns of critical transition intensities. In this study, the Sequential t-test Analysis of Regime Shifts (STARS) method proposed by Rodionov (2004) was employed to detect potential regime shifts in the alpine grassland ecosystem of Gannan Prefecture. The STARS method has the following advantages: (1) it can effectively identify abrupt changes in ecosystem states even in relatively short time series, thereby improving the reliability of detection results; and (2) unlike traditional time-series analysis methods that rely on long-term historical data and primarily identify changes after they have occurred, this method can provide early warning signals of potential regime transitions to a certain extent (Li, 2017). This method has been widely applied in studies of terrestrial ecosystem dynamics and climate change (Mei et al., 2010). The implementation steps of the STARS algorithm are as follows (Yang, 2019; Rodionov, 2004):

(1) Input time series: $X=\left(x_{1},x_{2},\ldots ,x_{n}\right)$;
(2) Set the window length L and significance level α (L = 5; α=0.05);
(3) Calculate the in-window variance ${\text{σ}}_{L}^{2}$;
(4) Compute the critical difference value: $diff=t_{\alpha }\sqrt{\frac{2{\text{σ}}_{L}^{2}}{L}}$;
(5) Calculate the current regime mean: $XR_{1}={\bar{X}}_{i,j}$;
(6) If *X_t_* > *XR*_1_ + *diff* or *X_t_*< *XR*_1_ − *diff*, proceed to candidate regime detection. Otherwise, include *X_t_* in the current regime interval, update *XR*_1_, and continue detection;
(7) Compute the regime shift index: $RSI=\sum \frac{X_{p}^{*}}{L_{\text{σL}}}\;$;
(8) A regime shift point is identified when RSI>0.

Fractional vegetation cover (FVC) and net primary productivity (NPP) were used as two integrated indicators to characterize the regime dynamics of the grassland ecosystem in Gannan Prefecture. RSI was calculated by accumulating normalized anomalies within each window to determine whether a candidate shift represented a statistically significant regime transition. Specifically, positive RSI values indicate that the cumulative anomaly exceeds the critical threshold, suggesting the occurrence of a regime shift. To further quantify the magnitude of regime shifts, RSI values were calculated on a pixel-by-pixel basis to characterize the intensity of regime shifts in the alpine grassland ecosystem.

#### 2.3.3 Ecological resilience calculation

Based on the identification of ecosystem regime shifts, this study further employed the classic “ball-and-cup” model to quantitatively assess ecosystem resilience (Yang, 2019; Sun et al., 2013; Holling, 1996). This model conceptualizes ecosystems as having multiple stable states (represented by “cups” or basins of attraction), with the current ecosystem state represented by a “ball” within one of these basins. Ecological resilience is defined as the maximum amount of disturbance an ecosystem can absorb while maintaining its original structure and function, before crossing a threshold (the edge of the cup) and shifting to an alternative stable state. Within a stable regime, the total cumulative interannual fluctuations of these indicators represent the total amount of disturbance absorbed by the ecosystem to maintain its current state. Therefore, higher cumulative fluctuations correspond to greater ecosystem resilience (i.e., the system can withstand larger disturbances without crossing a regime shift threshold), while smaller cumulative fluctuations indicate lower resilience and higher vulnerability to perturbation. The resilience index was calculated as follows (Yao et al., 2025; Walter et al., 2020; Kumar and Sharma, 2023):


$$R=\frac{\sum_{t_{1}}^{t_{2}-1}\Delta X_{t}}{T}$$

In this equation, R represents the ecological resilience of the grassland ecosystem under a stable regime; *t*_1_ denotes the initial year of a regime shift, and *t*_2_ denotes the subsequent regime shift year identified by the STARS algorithm. T = *t*_2_ − *t*_1_ represents the duration of the stable regime between two adjacent regime shifts, and Δ*X_t_* describes the annual disturbance intensity experienced by the ecosystem during the stable regime period, defined as the absolute value of the interannual difference in FVC or NPP values:


$$\Delta X_{t}=\left|X_{t+1}-X_{t}\right|$$

It should be noted that before regime shift detection and ecological resilience calculation, the annual FVC and NPP time-series datasets from 2001 to 2023 were smoothed using Gaussian filtering (bandwidth=5.0; kernel standard deviation=1.0) to reduce noise effects and abnormal fluctuations in remote sensing observations.

## 3 Results

### 3.1 Area change of alpine grassland in Gannan Prefecture

#### 3.1.1 Analysis of land cover area change

**Figure 2:**
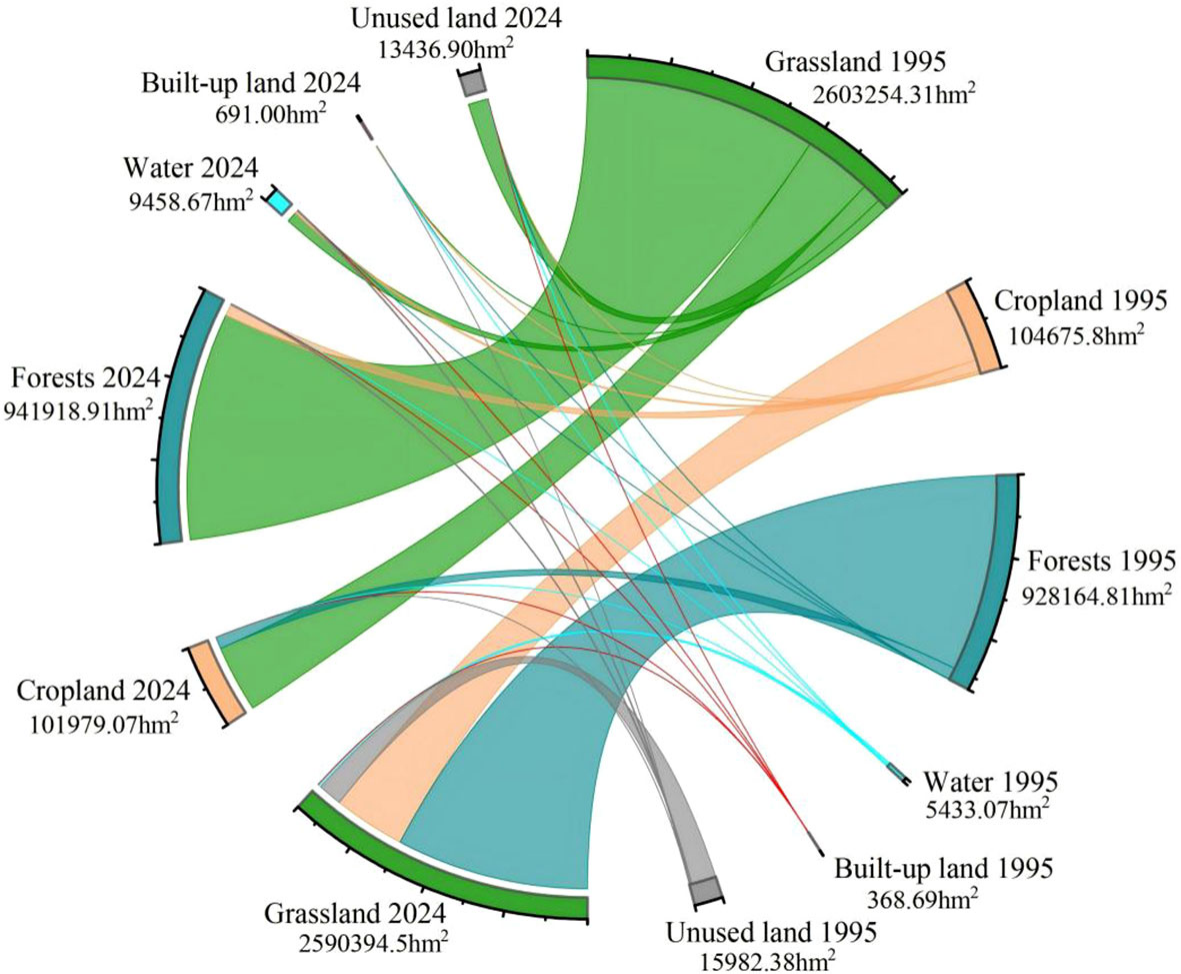
Land cover change in Gannan Prefecture between 1995 and 2024 (retaining only transition flows between different types, excluding stable flows within the same type; the width of the flow arrows represents the magnitude of land use conversion area).

**Figure 3:**
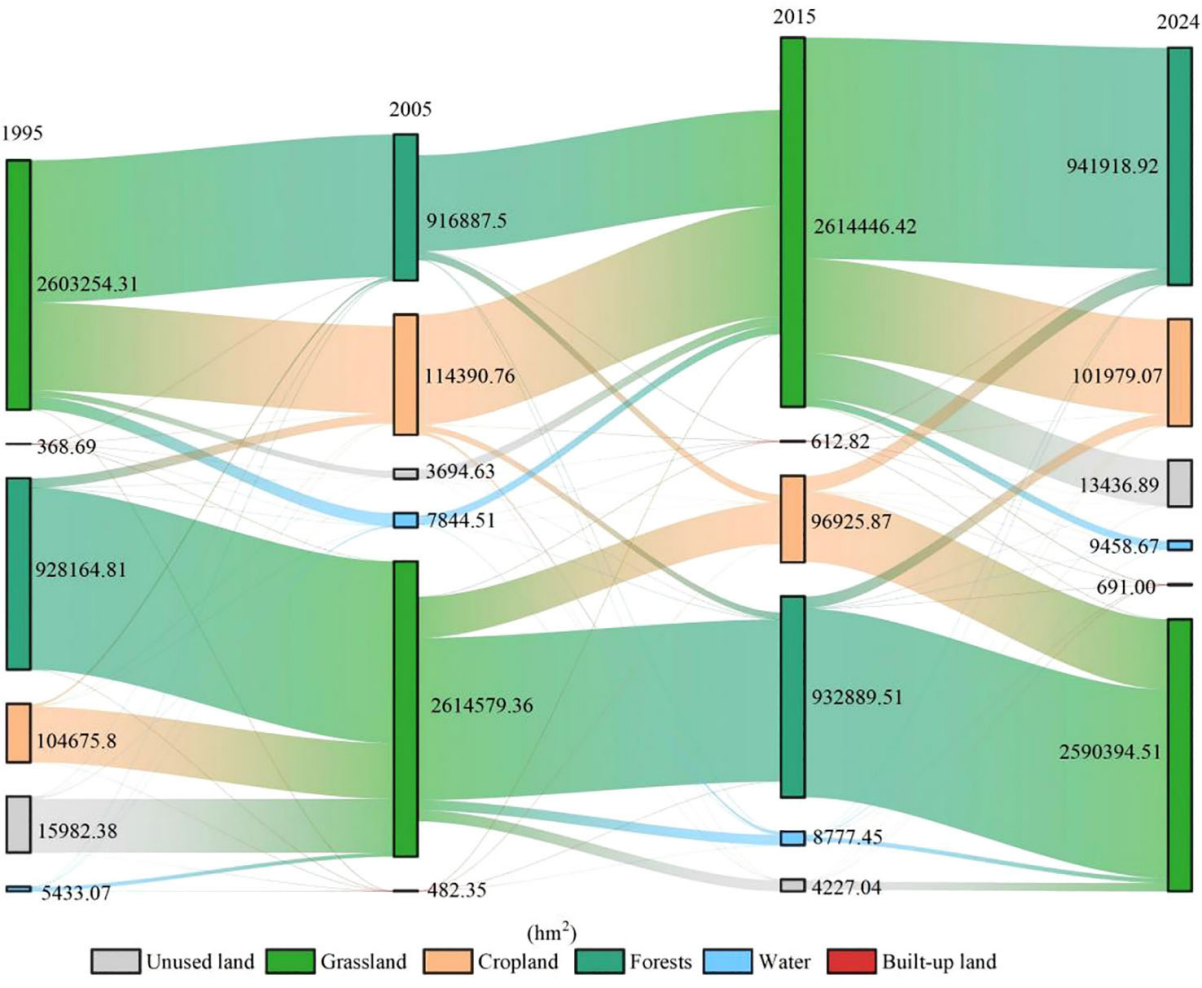
Land use transition flows (Sankey diagram) in Gannan Prefecture across different periods from 1995 to 2024 (retaining only transition flows between different types, excluding stable flows within the same type; Thicker flow bands indicate stronger land use transfer intensity).

To investigate grassland area changes in Gannan Prefecture from 1995 to 2024, this study utilized land cover data from four time points (1995, 2005, 2015, and 2024). Using ArcGIS 10.8 and Excel, spatial overlay and statistical analyses were conducted on land use types across different periods, and land use transition matrices were generated for four periods: 1995–2024, 1995–2005, 2005–2015, and 2015–2024 (**Figure 2**, **3**). Overall, the land use structure was dominated by grassland and forests, accounting for approximately 71% and 25% of the total area, respectively. Significant changes in land use structure occurred during 1995–2024, with decreases observed in grassland, cropland, and unused land. Specifically, grassland area decreased from 2603254.31 hm^2^ in 1995 to 2590394.50 hm^2^ in 2024, representing a reduction of 12,859.81 hm^2^ (approximately 0.5%), primarily due to conversion to forest land. Cropland area decreased from 104675.80 hm^2^ in 1995 to 101979.07 hm^2^ in 2024, a reduction of 2696.73 hm^2^ (approximately 2.6%), mainly converting to grassland and forests. Unused land also decreased from 15982.38 hm^2^ in 1995 to 13,436.90 hm^2^ in 2024, representing a reduction of 2545.48 hm^2^ (approximately 16.25%), primarily converting to grassland (9,370.83 hm^2^) and forest land (508.75 hm^2^). Conversely, forests, water, and built-up land exhibited varying degrees of expansion, particularly forest land, which increased by 13754.10 hm^2^ (1.5%) from 1995 to 2024 (**Figure 2**).

Between 1995 and 2005, grassland exhibited the largest area change among all land use types, with an annual net increase of 1,132.51 hm^2^, followed by cropland, which showed an annual net increase of 971.50 hm^2^. In contrast, forests and unused land experienced annual net decreases of 1,127.73 hm^2^ and 1,228.78 hm^2^, respectively, which were primarily converted to grassland. Unlike the land use transitions observed during 1995–2005, the period 2005–2015 was characterized by the greatest conversion in cropland, with an annual net decrease of 1,746.49 hm^2^, which was primarily converted to grassland and forests. During this period, forests exhibited an annual net increase of 1,600.20 hm^2^, while the changes in other land use types were relatively minor. From 2015 to 2024, grassland again showed the largest area change; however, unlike the 1995–2005 period, it exhibited a decrease, with an annual net decrease of 2672.4 hm^2^, which was primarily converted to forests and cropland. Meanwhile, forests and unused land increased by 1003.3 hm^2^/a and 1023.3 hm^2^/a, respectively (**Figure 3**).

#### 3.1.2 Characteristics of grassland area change

**Figure 4:**
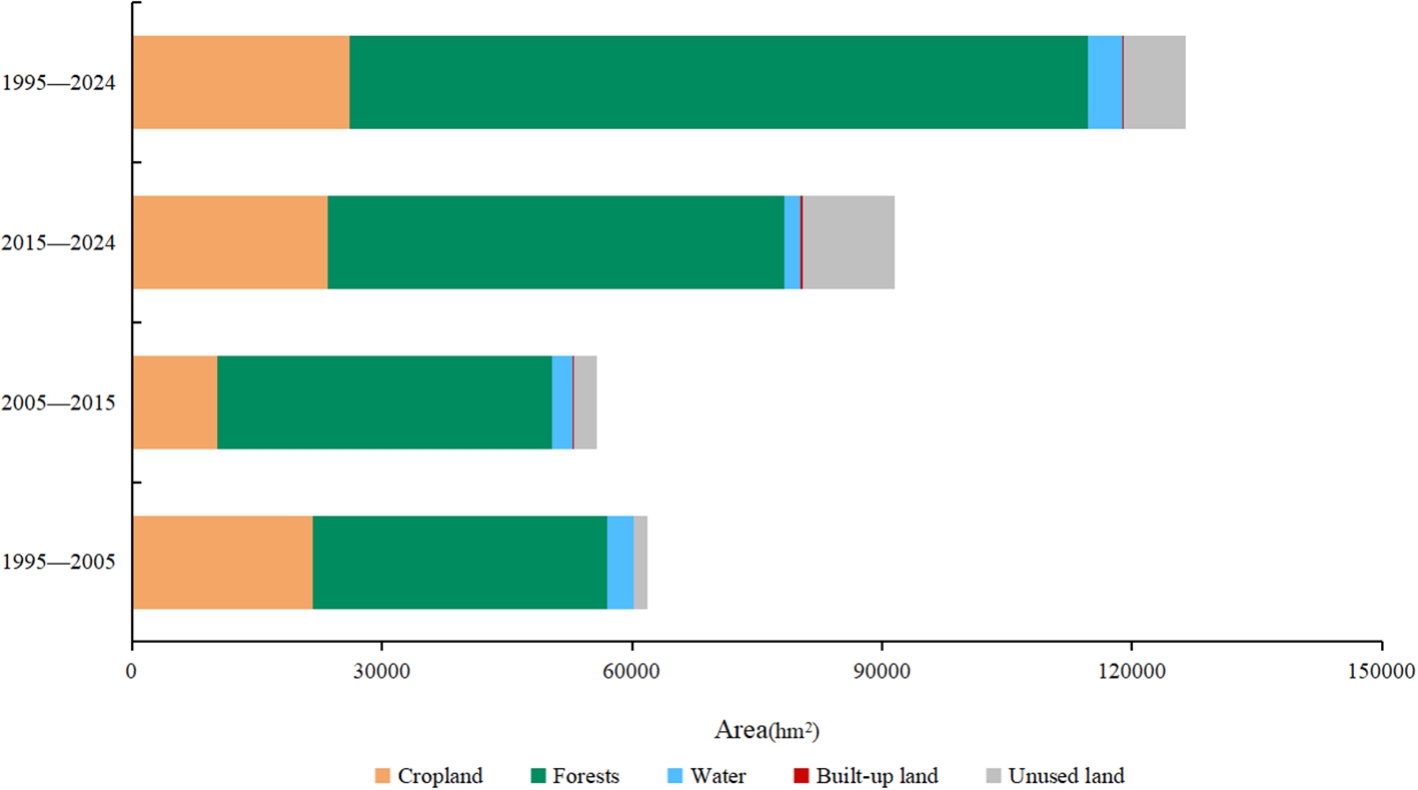
Grassland conversion area change.

**Figure 5:**
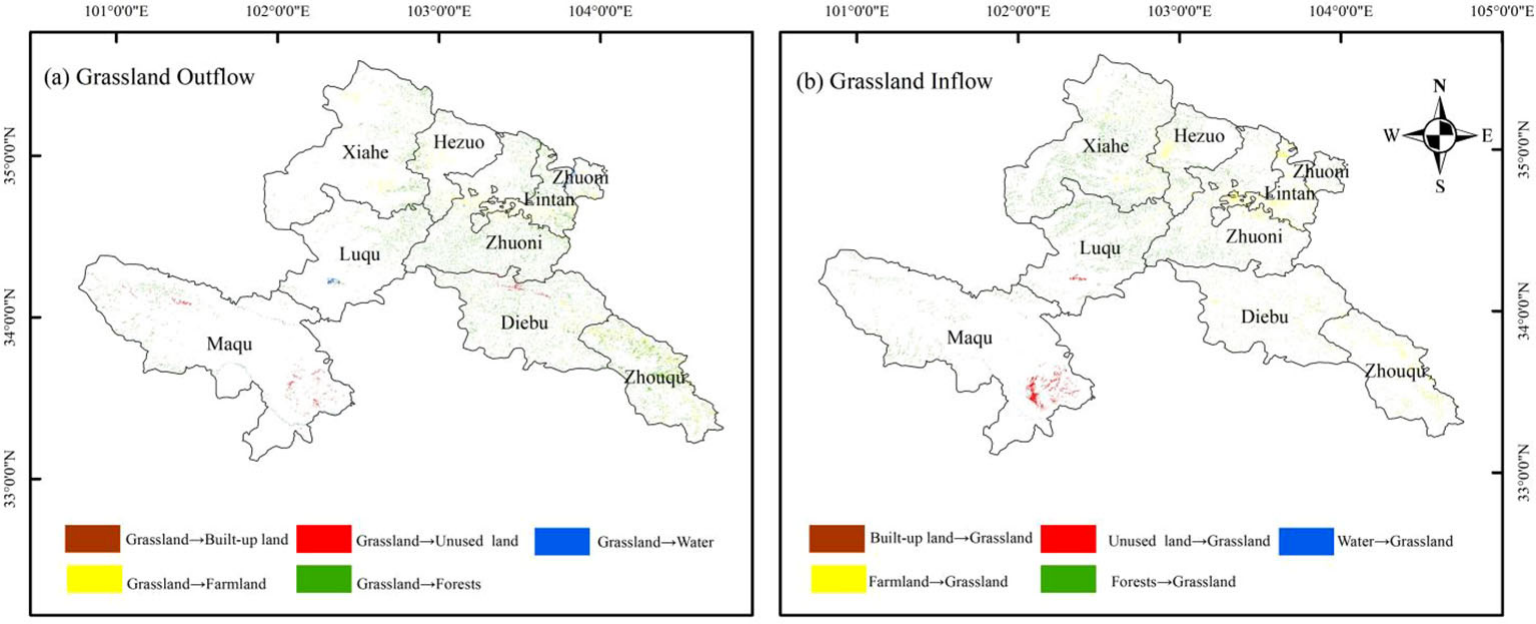
Spatial dynamics of grassland change in Gannan Prefecture from 1995 to 2024 [(a) grassland outflow areas; **(b)** grassland inflow areas].

In terms of grassland outflow across different periods, grassland was primarily converted to forests and cropland (**Figure 4**). To better illustrate the spatial characteristics of grassland change, the spatial patterns of grassland outflow (conversion from grassland) and inflow (conversion into grassland) were analyzed. From 1995 to 2024, grassland change occurred throughout the entire study region (**Figure 5**). In terms of spatial distribution, areas where grassland was converted to forests were mainly located in Zhuoni County, Diebu County, and Zhouqu County, covering approximately 88,600 hm^2^; Areas where grassland was converted to cropland were primarily distributed in Lintan County, Xiahe County, and Zhouqu County, with a total area of approximately 26,100 hm^2^. Areas where grassland degraded to unused land were mainly found in Maqu County, covering approximately 7,351.58 hm^2^. Regarding inflow, forest-to-grassland conversion accounted for the largest area (approximately 75,000 hm^2^), primarily distributed in Xiahe County and Luqu County; Cropland-to-grassland conversion was mainly observed in Lintan County, Hezuo City, and Zhouqu County, covering approximately 27,900 hm^2^. The area of unused land converted to grassland was larger than that of the area where grasslands degraded to unused land, mainly distributed in the eastern part of Maqu County, with an area of approximately 9,369.98 hm^2^.

### 3.2 Spatial-temporal characteristics of regime shift in alpine grassland

#### 3.2.1 Spatial-temporal characteristics of regime shift based on FVC

**Figure 6:**
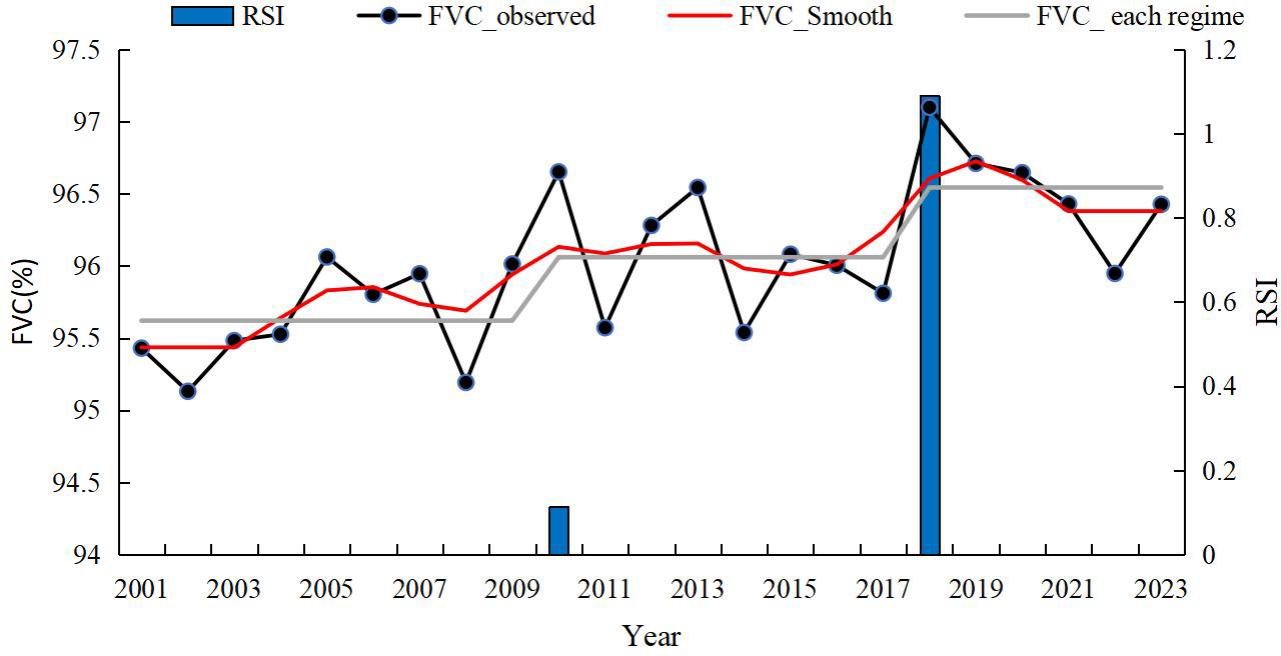
Time series of FVC and regime shift dynamics in alpine grassland of Gannan Prefecture from 2001 to 2023 (FVC_Smooth represents the smoothed FVC curve; RSI denotes the regime shift index).

The STARS algorithm identified two abrupt changes in FVC, occurring around 2010 and 2018 (**Figure 6**). The change around 2018 was the most significant, with a maximum RSI value of 1.09, while the shift around 2010 was relatively minor, with an RSI value of 0.11. Based on these transition points, the period from 2001 to 2023 can be divided into three regimes: 2001–2009, 2010–2017, and 2018–2023, with average FVC values of 95.62%, 96.06%, and 96.54%, respectively. During the first regime (2001–2009), a notable low value was observed around 2008. Around 2010, FVC exhibited an abrupt shift, entering a new regime characterized by a higher average FVC. This trend persisted until approximately 2018, when FVC experienced another abrupt shift, and the average FVC increased again.

**Figure 7:**
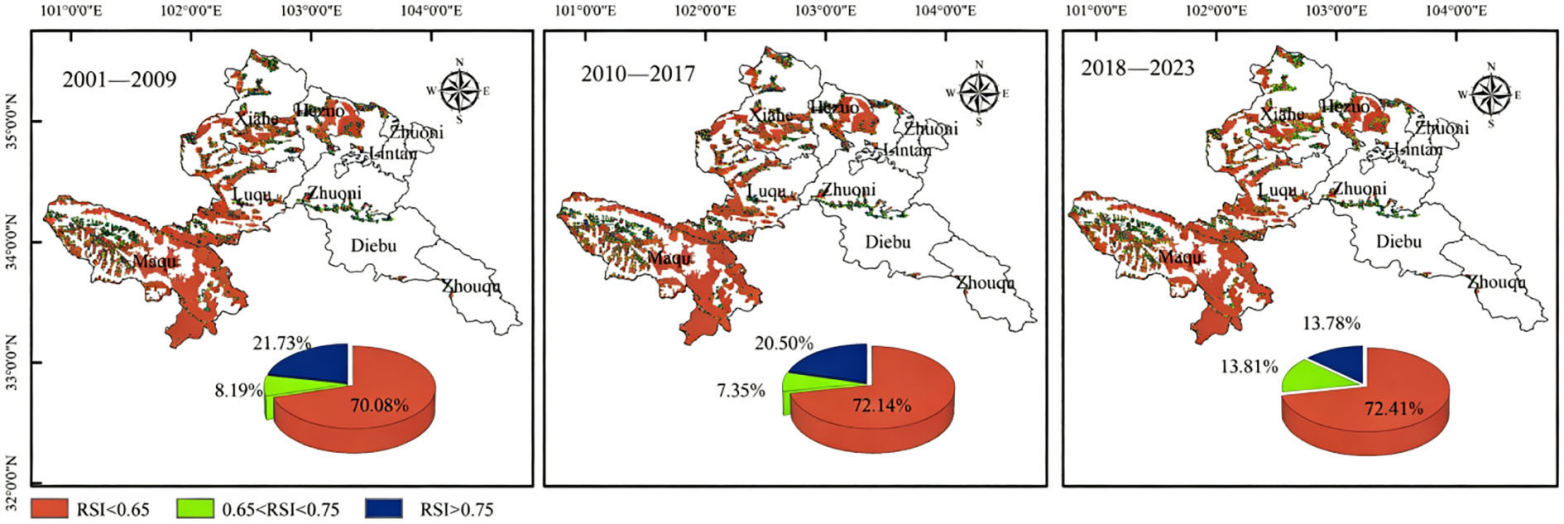
Spatial distribution of RSI in alpine grassland of Gannan Prefecture across different regime periods based on FVC (Pie chart showing the area proportions of RSI categories).

Based on pixel-scale RSI calculations for the FVC regime periods, RSI values were classified into three levels: weak (RSI< 0.65), moderate (0.65< RSI< 0.75), and strong (RSI > 0.75). Statistical analysis revealed that during 2001–2010, most alpine grassland areas in Gannan Prefecture exhibited relatively weak transition intensities, accounting for 70.08% of the total area, whereas areas with relatively strong transition intensities accounted for 21.73%, which were primarily distributed in the northwestern part of Maqu County. From 2010 to 2018, areas with moderate and strong transition intensities accounted for 7.35% and 20.50% of the region, respectively. Compared with the 2001–2010 period, the proportions of these two categories decreased, while the proportion of areas with weak transition intensity increased. During 2018–2023, the proportion of areas with RSI > 0.75 continued to decline, with reductions mainly observed in Xiahe County and Hezuo City in northern Gannan Prefecture (**Figure 7**).

#### 3.2.2 Spatial-temporal characteristics of regime shift based on NPP

**Figure 8:**
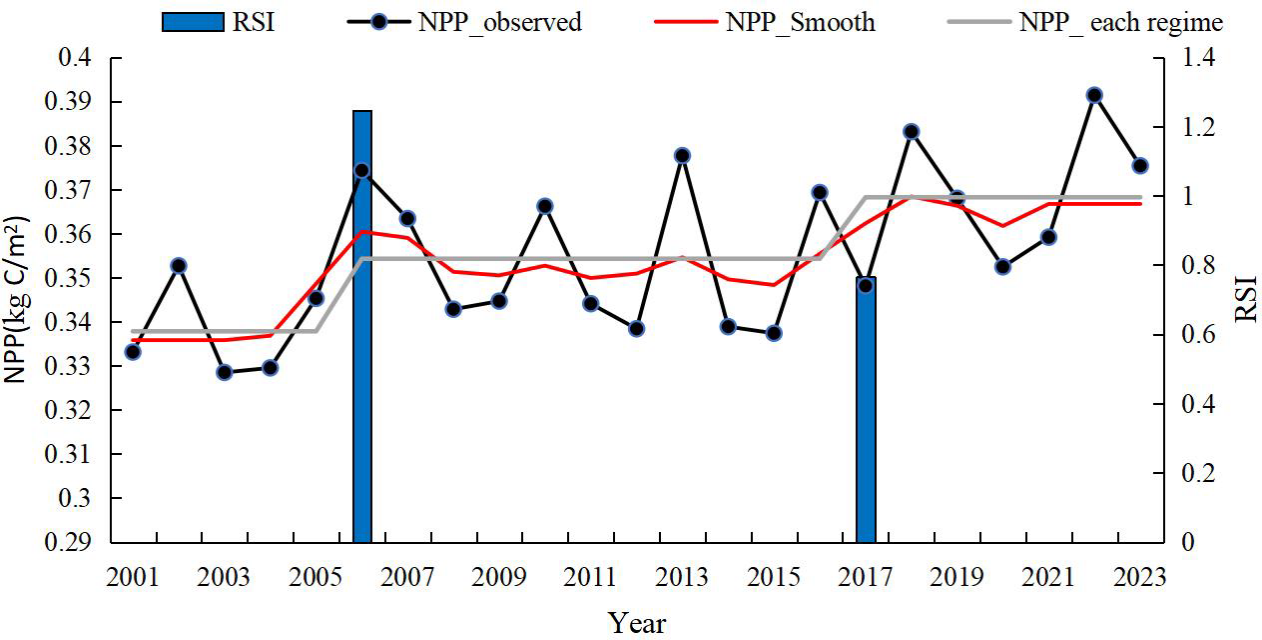
Time series of NPP and regime shift dynamics in alpine grassland of Gannan Prefecture from 2001 to 2023 (NPP_Smooth represents the smoothed NPP curve; RSI denotes the regime shift index).

Based on the analysis of NPP from 2001 to 2023, two abrupt regime shifts were identified in the alpine grasslands of Gannan Prefecture (**Figure 8**), occurring in 2006 and 2017. Accordingly, the study period can be divided into three regimes: 2001–2005, 2006–2016, and 2017–2023, with corresponding mean NPP values of 0.34, 0.35, and 0.37 kg C m^-2^, respectively. The regime shift around 2006 was particularly significant, with an RSI value of 1.25.

**Figure 9:**
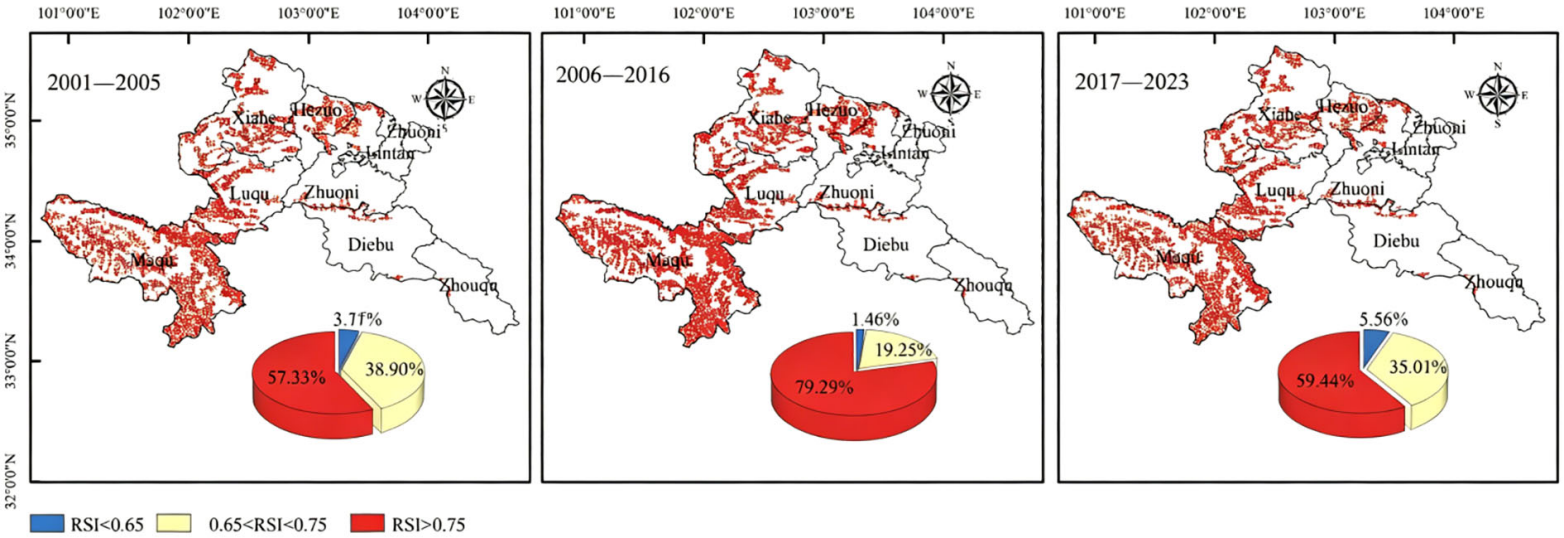
Spatial distribution of RSI in alpine grassland of Gannan Prefecture across different regime periods based on NPP (Pie chart showing the area proportions of RSI categories).

Spatially, the RSI values of NPP-based regime shifts showed greater magnitudes than those derived from FVC, with most areas having RSI > 0.75. Specifically, during 2001–2005, moderate and strong transition intensities accounted for 38.90% and 57.33% of the total area, respectively. From 2006 to 2016, the proportion of areas with strong transition intensity (RSI > 0.75) increased to 79.29%, while that of areas with moderate transition intensity decreased to 19.25%. In other words, following the 2006 regime shift, most regions exhibited increased RSI values, with the most intense transitions concentrated in Maqu County. During 2017–2023, RSI values decreased again, with strong transition intensity observed in 59.44% of the total area (**Figure 9**).

### 3.3 Resilience variation characteristics of alpine grassland

Across different regimes, ecosystems experience varying levels of external disturbances, and their resilience correspondingly changes. Therefore, the resilience of a given regime is defined as the ratio of the total disturbance absorbed by the grassland during that regime to the duration of the regime, representing the average rate at which the system returns to its regime during fluctuations within the regime.

#### 3.3.1 Characteristics of ecological resilience changes based on FVC

**Figure 10:**
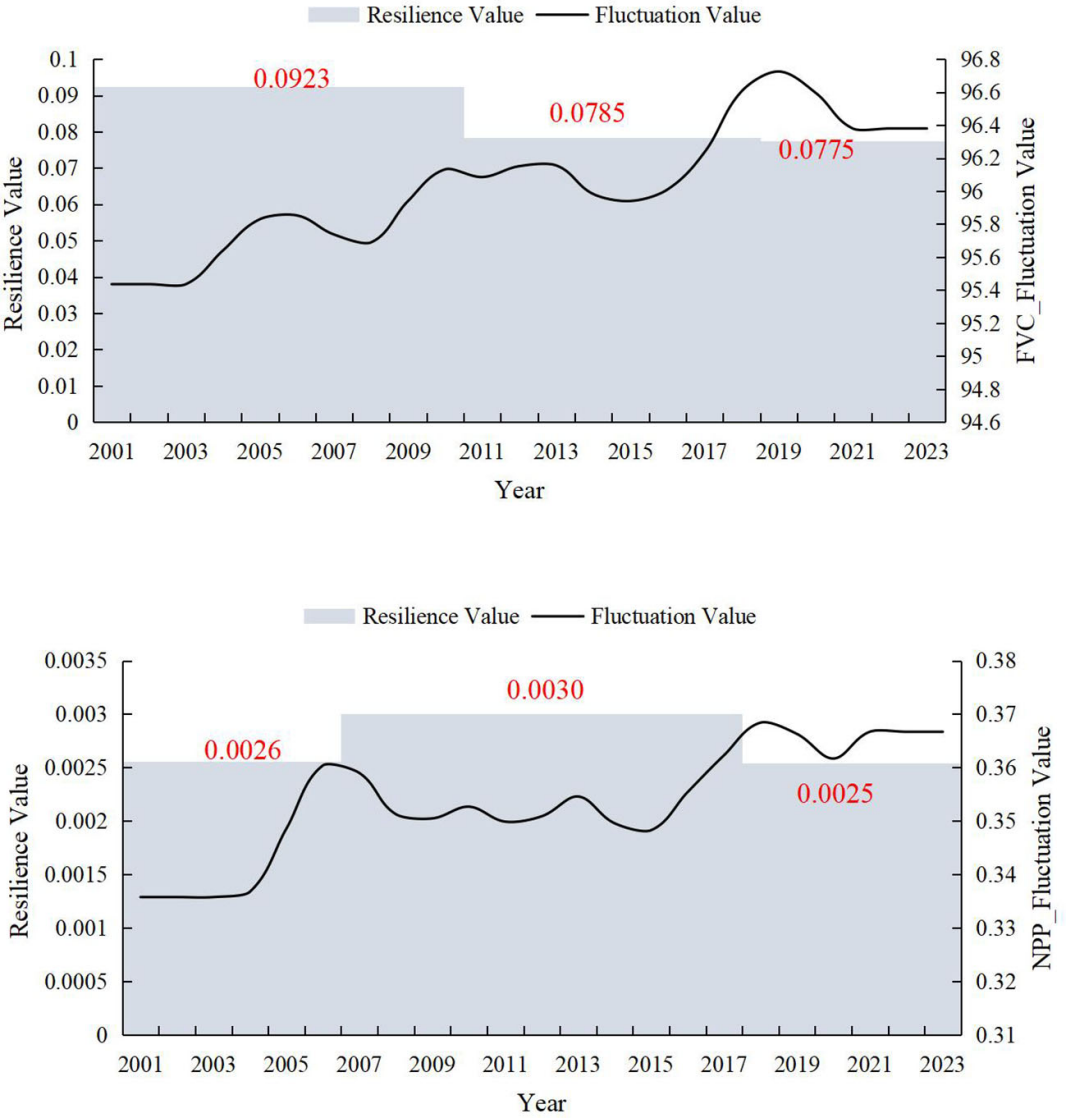
Variation of FVC and precipitation (left), NPP and temperature (right) in alpine grassland of Gannan Prefecture.

The ecological resilience calculated based on FVC indicated that the alpine grasslands in Gannan Prefecture exhibited an overall decreasing trend that gradually stabilized during 2001–2023 (**Figure 10**). Overall, the resilience value decreased from 0.0923 to 0.0775, representing a decline of 16.03%. During the transition from the first stable regime (2001–2009) to the second stable regime (2010–2017), FVC increased whereas ecological resilience decreased by 14.95%. From the second stable regime (2010–2017) to the third stable regime (2018–2023), ecological resilience further declined slightly; however, the magnitude of change became noticeably weaker and gradually approached a relatively stable state. These results indicate that the alpine grassland ecosystem gradually entered a new relatively stable regime after experiencing two regime shifts.

#### 3.3.2 Characteristics of ecological resilience changes based on NPP

The ecological resilience calculated based on NPP exhibited a trend of initial increase followed by subsequent decline during 2001–2023 (**Figure 10**). Overall, the resilience value decreased slightly from 0.0026 to 0.0025, representing an overall decline of 3.85%. Both FVC- and NPP-based analyses identified two major regime shifts in the alpine grassland ecosystem, and the detected transition periods showed certain similarities. However, the ecological resilience dynamics characterized by the two indicators differed. Specifically, FVC-based resilience exhibited an overall decreasing trend, whereas NPP-based resilience showed a pattern of initial increase followed by subsequent decline.

### 3.4 Impact of climate change on the regime shifts of alpine grassland

To explore the relationships between climate factors and alpine grassland regime dynamics, Spearman correlation analysis was applied to the 2001–2023 time series to quantitatively assess the associations between FVC/NPP and precipitation/temperature (**Table 2**). The results showed that FVC was significantly positively correlated with precipitation (Spearman correlation coefficient = 0.50, P< 0.05), indicating that increased precipitation was associated with enhanced regional vegetation coverage, whereas no significant correlation was found between FVC and temperature. In contrast, NPP showed a significantly positive correlation with temperature (Spearman correlation coefficient = 0.47, P< 0.05) but no significant correlation with precipitation. These findings indicate that regime dynamics of alpine grasslands in Gannan Prefecture were jointly associated with variations in precipitation and temperature.

**Table 2:** Spearman correlation analysis between FVC, NPP, and precipitation and temperature.

Variable	Factor	Spearman correlation coefficient	P	Significance
FVC	Precipitation	0.4977	0.0255	Significant (p<0.05)
FVC	Temperature	0.2165	0.3591	Non-significant (p≥0.1)
NPP	Precipitation	0.0647	0.7865	Non-significant (p≥0.1)
NPP	Temperature	0.4662	0.0383	Significant (p<0.05)

**Figure 11:**
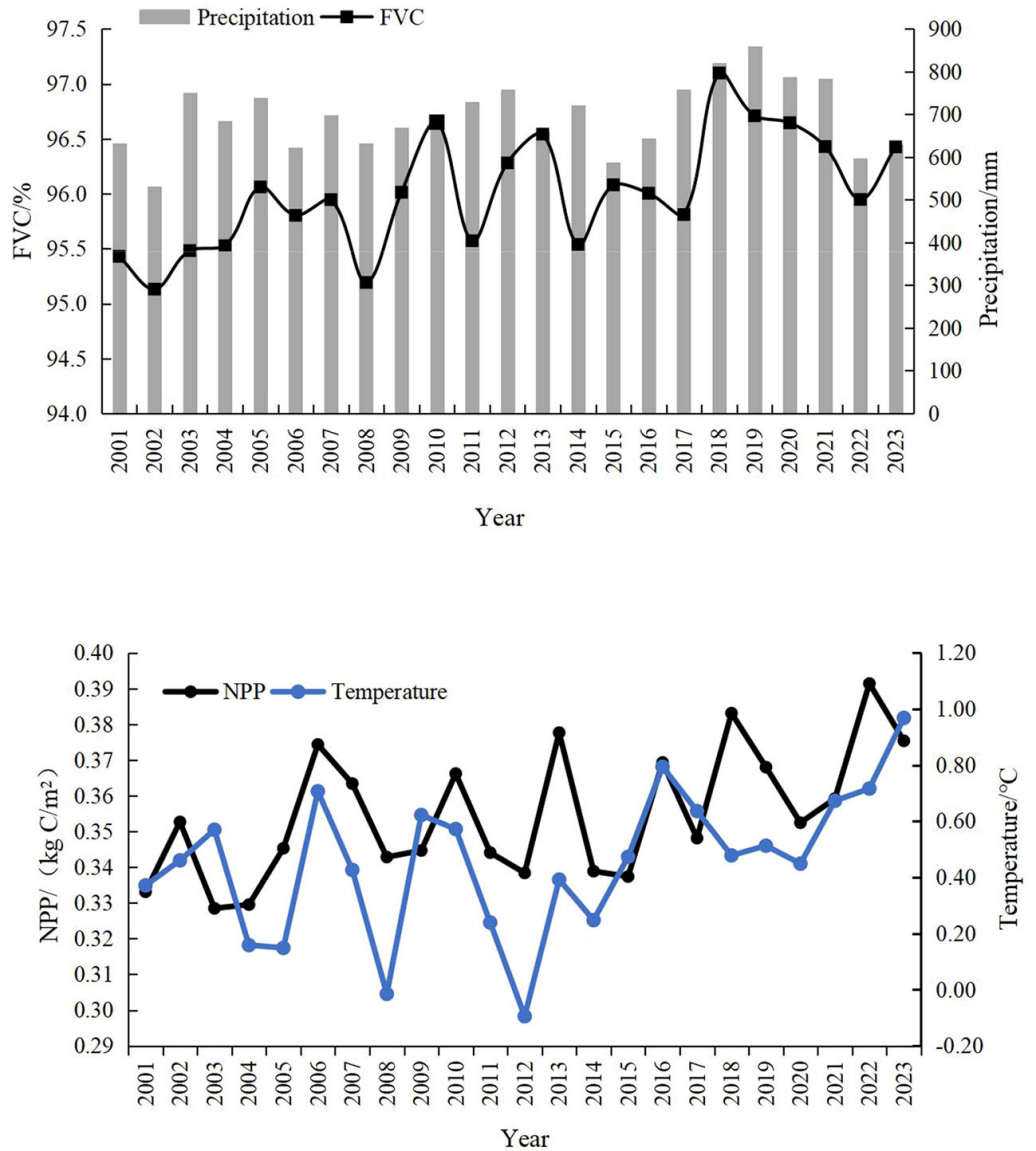
Smoothed fluctuation values of FVC (left) and NPP (right) and their resilience changes in alpine grassland of Gannan Prefecture from 2001 to 2023.

Further analysis of the temporal variation patterns indicated that FVC changes were temporally consistent with precipitation variability and may exhibit a potential lagged response (**Figure 11**). Specifically, the first regime shift in FVC occurred in 2010 following a relatively humid period from 2003 to 2010. Similarly, although precipitation increased in 2003, it did not immediately trigger changes in FVC. The second regime shift of FVC occurred in 2018. After the initial transition in 2010, high precipitation levels persisted from 2011 to 2014, and although precipitation decreased in 2015, FVC did not shift until three years later, demonstrating a lag effect. These temporal differences suggest that precipitation may influence alpine grassland vegetation cover through cumulative ecological processes rather than immediate responses.

In contrast, NPP variations showed relatively consistent temporal patterns with annual mean temperature. The two regime shifts of NPP detected in 2006 and 2017 coincided with periods of relatively rapid temperature increase. This finding suggests that temperature variability may be closely associated with interannual changes in vegetation productivity and the regime shift process of alpine grasslands.

## 4 Discussion

### 4.1 Spatiotemporal evolution of land use patterns

This study analyzed the spatiotemporal evolution of land use patterns in Gannan Prefecture from 1995 to 2024 and conducted a phased analysis of their transformation processes. Particular emphasis was placed on the dynamic changes in grassland area over the past 29 years. The results indicate that during 1995–2005, forests and unused land were primarily converted to grassland, a phenomenon that may be associated with the implementation of ecological restoration policies such as farmland return programs and the development of livestock husbandry. Between 2005 and 2015, cropland was mainly transformed into grassland and forests, a structural adjustment that might be attributed to the sustained advancement of ecological protection policies and forestry initiatives, including afforestation, farmland return to grassland, and farmland return to forests. Simultaneously, agricultural restructuring may have facilitated the conversion of cultivated land to other uses. During 2015–2024, the most significant changes occurred in grassland area, which exhibited an overall decreasing trend. This reduction may be attributed to the continued implementation of ecological restoration measures, including afforestation and forest conservation through enclosure, which promoted the natural succession or the artificial conversion of grassland to forest. Additionally, regional socioeconomic development has driven the reclamation of underutilized land through land consolidation and soil improvement projects, further influencing land use pattern transformations.

### 4.2 Ecological resilience dynamics of alpine grasslands

Using long-term remote sensing datasets, this study quantitatively analyzed regime shifts and ecological resilience of grassland ecosystem, emphasizing the critical role of ecological resilience in maintaining ecosystem stability and promoting sustainable development. By characterizing the spatiotemporal variations in grassland ecological regimes and resilience, this research provides a scientific reference for the assessment and management of grassland ecosystems in other regions (Qu et al., 2025). The results indicated that the ecological resilience dynamics of alpine grasslands derived from FVC and NPP exhibited certain differences. Specifically, the ecological resilience calculated based on FVC showed an overall decreasing trend that gradually stabilized, whereas the resilience derived from NPP exhibited a pattern of initial increase followed by subsequent decline. These findings suggest that the ecological stability of alpine grasslands in Gannan Prefecture experienced stage-dependent variations during the study period, and that different ecological indicators responded differently to ecosystem stability. During the early stage of the study period, both FVC and NPP generally increased, while ecological resilience remained at relatively high levels, indicating that under the background of climate warming and humidification, vegetation growth conditions in alpine grasslands improved and the adaptability and stability of the ecosystem in response to external disturbances were enhanced. This beneficial effect was most pronounced in dominant *Kobresia* and *Stipa* species of Gannan alpine meadows, which have evolved dense fibrous root systems in the top of soil in study area. These root networks enable rapid water uptake and storage, effectively buffering aboveground growth against short-term precipitation variability—a key adaptive strategy in the highly variable alpine environment (Li et al., 2024). During this period, precipitation generally exhibited an increasing trend, while temperature increased moderately, which may have contributed to vegetation recovery and productivity improvement in alpine grasslands (**Figure 11**). In addition, these changes may also be closely associated with the long-term implementation of ecological restoration policies, such as afforestation and grazing management, which have improved vegetation structure and ecosystem stability (Wang et al., 2025a; Chen et al., 2026).

However, after 2017–2018, although FVC and NPP remained at relatively high levels, ecological resilience showed a declining trend. These findings suggest that increases in vegetation cover and productivity may not necessarily correspond to continuous enhancement of ecosystem stability. During the study period, the annual mean temperature increased continuously after 2017 and reached 0.968 °C in 2023, which was significantly higher than that in the earlier years. Meanwhile, although precipitation remained relatively high overall, interannual fluctuations became more pronounced, especially with a substantial decline in precipitation in 2022 (**Figure 11**). In Gannan alpine meadows, such fluctuations likely amplified soil water deficits due to shallow soils and high evapotranspiration (Zhang et al., 2025c), reducing the ability of dominant graminoid species to maintain stable canopy cover, thereby explaining the sharper decrease in FVC-based resilience compared with NPP-based resilience. A study has found that under continuous warming conditions, potential increases in evapotranspiration in alpine grassland ecosystems may alter the regional water–heat balance, thereby enhancing ecosystem sensitivity to climate variability and extreme climatic events and ultimately leading to decreased ecological resilience (Bai et al., 2025).

In addition, alpine grassland ecosystems are highly sensitive to climate change, and vegetation structure recovery is not always synchronized with improvements in ecosystem stability. Although the warming and humidification trend promoted increases in vegetation cover and productivity, continuous warming may gradually weaken the internal stability of the ecosystem and increase the risk of the ecosystem approaching potential regime shift thresholds (Wu et al., 2020). In this study, the decline in ecological resilience detected based on FVC (16.03%) was considerably greater than that detected based on NPP (3.85%), indicating that vegetation structure in alpine grasslands may be more sensitive to climate change and environmental fluctuations than ecosystem function. Therefore, rather than simply pursuing higher vegetation cover, maintaining an appropriate and stable vegetation structure may be more beneficial for the long-term stability and sustainable development of alpine grassland ecosystems. These findings are consistent with previous studies indicating that moderate vegetation cover is more conducive to maintaining ecosystem stability (Huang et al., 2026). Compared with previous studies that mainly evaluated vegetation change using a single indicator, this study demonstrates that structural and functional indicators may exhibit different resilience trajectories under climate change. Although both FVC and NPP detected similar regime shift periods, their resilience trends differed substantially, suggesting that vegetation greening does not necessarily indicate synchronous enhancement of ecosystem stability. This highlights the importance of integrating multiple ecological indicators when assessing ecosystem regime dynamics and resilience in alpine grasslands. Therefore, future grassland management should focus not only on increasing vegetation cover and productivity, but also on maintaining ecosystem stability and resilience under climate change.

### 4.3 Climatic drivers and ecological mechanisms of regime shifts

The differential responses of FVC and NPP to climatic factors reflect distinct ecological mechanisms underlying alpine grassland dynamics. Specifically, FVC appeared to exhibit a potential lagged response to precipitation. This lag can be explained by the interaction between shallow alpine soils with limited water storage capacity and the slow canopy expansion of dominant species (e.g., *Kobresia* meadows). In alpine ecosystems, precipitation does not immediately translate into vegetation cover changes; instead, it influences soil water availability, which subsequently regulates plant growth over time (Wang et al., 2025b). In contrast, NPP showed relatively synchronous variation patterns with temperature, indicating that thermal conditions may play an important role in regulating photosynthetic activity and carbon assimilation in these high-altitude ecosystems. Low temperatures directly constrain enzymatic activity and cell division rates in dominant alpine grasses, while warming extends the growing season length and enhances photosynthetic efficiency. This explains why NPP responded more immediately to interannual temperature fluctuations (Nam and Ferrarezi, 2026). The findings of this study are consistent with previous research highlighting the dominant role of precipitation in controlling vegetation cover in alpine and semi-arid regions, while temperature primarily regulates ecosystem productivity (Xu et al., 2025; Zhuo et al., 2025; Hou and Yu, 2025). For example, Zhuo et al. (2025) reported that regional vegetation recovery along the agro-pastoral ecotone of Gansu Province were strongly influenced by precipitation variability, whereas Hou and Yu (2025) emphasized the direct impact of atmospheric aridity conditions on vegetation productivity changes. Compared with these studies, the present research further reveals the regime shift characteristics and resilience dynamics of alpine grasslands in Gannan Prefecture, providing a more comprehensive understanding of ecosystem stability under climate change. However, the factors influencing regime shifts and resilience changes in grasslands of Gannan Prefecture are diverse and complex, including not only precipitation and temperature but also evapotranspiration, soil types, population density, and other variables (Chu et al., 2026). Therefore, it is necessary to further identify the key driving factors and quantitatively assess their relative contributions to changes in grassland ecological resilience.

### 4.4 Limitations

In this study, two indicators-fractional vegetation cover (FVC) and net primary productivity (NPP) were selected to represent vegetation structure and ecological function, respectively, and were jointly used to characterize regime shifts in grassland ecosystems. However, regime shifts in grassland ecosystems are not only reflected in vegetation cover and productivity but are also closely associated with multiple factors, including soil properties, biodiversity, and other ecological determinants. Therefore, future research should integrate multi-source remote sensing data with multi-indicator comprehensive approaches to further explore the variation characteristics of ecological regimes and resilience in grassland ecosystems.

## 5 Conclusion

1) The land use structure of Gannan Prefecture is dominated by grasslands and forests. From 1995 to 2024, grassland area decreased by approximately 0.5%, primarily converting to forests, mainly distributed in Zhuoni County, Diebu County, and Zhouqu County, covering approximately 88,600 hm^2^. In terms of conversions to grassland, the area converted from forests to grassland (approximately 75,000 hm^2^) exceeded that from other land use types and was mainly located in Xiahe County and Luqu County. Land use changes varied across different periods.

2) Based on structural (FVC) and functional (NPP) indicators, similar results were obtained in detecting the spatiotemporal characteristics of regime shifts and changes in ecosystem resilience of alpine grasslands in Gannan Prefecture during 2001-2023. Using FVC as the regime variable, two regime shifts were identified in 2010 and 2018, with the strongest intensity occurring in 2018. Similarly, using NPP as the regime variable, two regime shifts were detected in 2006 and 2017, with the most significant change occurring around 2006.

3) The ecological resilience dynamics of alpine grasslands in Gannan Prefecture characterized by FVC and NPP exhibited different patterns. Specifically, ecological resilience based on FVC decreased by 16.03%, whereas that based on NPP decreased by 3.85%.

4) Both temperature and precipitation were associated with regime dynamics of alpine grasslands in Gannan Prefecture. FVC-based regime shift suggested a possible lagged association with precipitation, whereas NPP-based regime shifts exhibited relatively synchronous patterns with temperature variability.

## Data Availability

The raw data supporting the conclusions of this article will be made available by the authors, without undue reservation.
